# Metformin-Encapsulated Liposome Delivery System: An Effective Treatment Approach against Breast Cancer

**DOI:** 10.3390/pharmaceutics11110559

**Published:** 2019-10-28

**Authors:** Snehal K. Shukla, Nishant S. Kulkarni, Amanda Chan, Vineela Parvathaneni, Pamela Farrales, Aaron Muth, Vivek Gupta

**Affiliations:** 1 Department of Pharmaceutical Sciences, College of Pharmacy and Health Sciences, St. John’s University, Queens, NY 11439, USA; snehal.shukla16@stjohns.edu (S.K.S.); nishant.kulkarni16@stjohns.edu (N.S.K.); Vineela.parvathaneni16@my.stjohns.edu (V.P.); pamela.farrales17@my.stjohns.edu (P.F.); mutha@stjohns.edu (A.M.); 2 Department of Biological Sciences, College of Liberal Arts and Sciences, St. John’s University, Queens, NY 11439, USA; amanda.chan16@my.stjohns.edu

**Keywords:** metformin, AMPK activation, breast cancer, liposomes, 3D spheroids

## Abstract

This study aimed at developing metformin hydrochloride (Met) encapsulated liposomal vesicles for enhanced therapeutic outcomes at reduced doses against breast cancer. Liposomal Met was prepared using thin-film hydration through various loading methods; passive loading, active loading, and drug-loaded lipid film. The drug-loaded film method exhibited maximum entrapment efficiency (~65%) as compared to active loading (~25%) and passive loading (~5%) prepared Met-loaded liposomes. The therapeutic efficacy of these optimized liposomes was evaluated for cellular uptake, cytotoxicity, inhibition of metastatic activity, and apoptosis-inducing activity. Results demonstrated significantly superior activity of positively charged liposomes resulting in reduced IC_50_ values, minimal cell migration activity, reduced colony formation, and profound apoptosis-induced activity in breast cancer cells as compared to Met. The anti-tumor activity was investigated using a clinically relevant in vitro tumor simulation model, which confirmed enhanced anti-tumorigenic property of liposomal Met over Met itself. To the authors’ knowledge, this is the first report of Met-loaded liposomes for improving the efficacy and therapeutic effect of Met against breast cancer. With the results obtained, it can be speculated that liposomal encapsulation of metformin offers a potentially promising and convenient approach for enhanced efficacy and bioavailability in breast cancer treatment.

## 1. Introduction

Breast cancer is the most common type of cancer found worldwide with high incidence rates making it a huge burden on global mortality [1]. Specifically, breast cancer accounts for ~25% of newly diagnosed cancer cases occurring globally [1,2]. According to a 2019 report from the American Cancer Society, about 3 million people in the United States are reported to have a family history of breast cancer. Furthermore, 12% of all women will be diagnosed with breast cancer in their lifetime [3]. Breast cancer is known to be a heterogenous and complex disease, classified based on expression of receptors; estrogen receptors (ER) positive, progesterone receptors (PR) positive, human epidermal growth factor receptor (HER2) amplified breast cancer, or triple negative breast cancer (TNBC) [4,5]. While most breast cancer subtypes can be specifically targeted, TNBC presents challenges to existing therapies as it expresses no receptors and therefore has been the most difficult to treat [5,6]. The existing treatment regimen constitutes primarily of chemotherapy, radiation therapy, surgery, or chemo-radio therapy. Existing therapies also combat issues such as severe adverse effects, resistance development, and the requirement of palliative care post-treatment. Therefore, there is a dire need to discover or develop new and effective approaches for breast cancer treatment, which are safe and possess less off-target side effects. Several studies have linked 5’ adenosine monophosphate (AMP)-activated protein kinase (AMPK) (an enzyme regulating cellular metabolism and homeostasis) activator molecules to demonstrating efficacy in the treatment of various breast cancer subtypes, including TNBC [4,5,7,8]. Therefore, we decided to evaluate the efficacy of Met, an AMPK activator, on the treatment of breast cancer via encapsulation in a lipid delivery carrier.

Metformin hydrochloride (Met), an FDA-approved anti-diabetic drug, has been commonly prescribed as a first-line treatment of Type-II diabetes mellitus. Met was first discovered in 1922 by Werner and Bell [9] and is the most prescribed anti-hyperglycemic drug globally; which subsequently led to it being listed in the World Health Organization’s List of Essential Medicines 2011 [10]. Met has been explored by various researchers for its efficacy in several diseases, such as malaria [11], influenza [12], and polycystic ovarian disease [13]. Met has also been found to decrease the risk of several cancers, such as colorectal, pancreatic, breast, prostate, and non-small cell lung cancer in patients taking metformin as compared to non-metformin users [14,15]. In breast cancer, Met’s mechanism of action has been attributed to the activation of AMPK, which is a metabolic sensor involved in regulating homeostasis in cellular activities [16]. AMPK activation triggers the inhibition of mammalian target of rapamycin (mTOR) downstream signaling along with down-regulation of Ras-mitogen-activated protein kinase (MAPK) signaling pathway, which inhibits tumorigenesis; inhibition of cellular proliferation and metastasis [16,17]. Additionally, Met has shown to reverse multi-drug resistance (MDR), a major limitation observed with most chemotherapy treatments. Reversal of MDR in breast cancer is marked by downregulation of multidrug resistance 1 (MDR1) gene transcription and P-glycoprotein (P-gp) expression upon AMPK activation [18,19]. Met has also demonstrated an ability to reduce obesity, uphold lipid profiles, as well as reduce incidences of fatty liver and cardiovascular diseases [17]. Due to its safer profile, anti-tumor properties, and valuable clinical effects, Met is a potential candidate for an alternative treatment approach for breast cancer.

Met has been investigated clinically in breast cancer treatment as a combination therapy with other chemo-therapeutic drugs or adjuvants [14,20,21]. It requires being used in a combination therapy approach due to its high dose requirement (~1500–1800 mg/day) when used as single agent for treatment [21,22,23]. The required high dose is known to impact the therapeutic efficacy of Met due to dose-related gastrointestinal side effects, such as nausea, diarrhea, and abdominal pain along with lactic acidosis [20,22]. In a clinical trial performed to assess the effects of Met against breast cancer, ~12.5% of the patient population were found to be affected by gastrointestinal side effects, resulting in withdrawal from the studies [21]. Despite possessing both economic and therapeutic benefits, Met is limited for its use in breast cancer therapy as a single agent. Hence, developing a delivery system, which will exhibit efficacy at reduced doses and represent a potential strategy to overcome Met limitations while establishing it as a repurposed anticancer agent, is important.

Liposomes have been considered an effective carrier system for drugs requiring dose intensification due to their inherent ability to encapsulate drugs irrespective of their aqueous solubility [24]. These kinetically entrapped systems are known to possess superior stability along with other helpful properties such as biodegradability and biocompatibility due to its composition mimicking the cell membrane and its enhanced safety [25]. In addition, liposomes are known for offering versatility in terms of particle size as well as its physical parameters, such as membrane lipid packing, steric stabilization, surface charge, and desired route of administration for wide range application [26]. In recent years, the development of positively charged (cationic) liposomes has gained importance for the delivery of anticancer drugs due to their enhanced penetration in tumors and accumulation in newly formed angiogenic vessels [27]. Therefore, designing a cationic liposomal delivery system for Met may prove to be a promising strategy for breast cancer treatment with enhanced therapeutic efficacy at a reduced dose.

In this study, we aim to develop lipid-based nanocarriers for the delivery of Met to enhance breast cancer treatment. To the authors’ knowledge, this is one of the first studies reported to explore the potential of liposomal encapsulated Met for breast cancer therapy. We hypothesize that entrapment of Met in liposomes will address the issues of a high dose requirement, along with poor tumor accumulation, which will result in the development of an effective therapy with improved therapeutic outcomes.

## 2. Materials and Methods

### 2.1. Materials

1,2-dipalmitoyl-*sn*-glycero-3-phosphocholine (DPPC) and cholesterol were purchased from Echelon Biosciences (Salt Lake City, UT, USA). 1,2-dioleoyl-3-trimethylammonium-propane (chloride salt) (DOTAP) was obtained from Avanti Polar Lipids (Alabaster, AL, USA). Metformin hydrochloride (Met) was purchased from Cayman Chemical Company (Ann Arbor, MI, USA). Phosphate Buffer saline (PBS) pH 7.5 and HPLC grade solvents—acetonitrile and water—were procured from Fisher Bioreagents (Fisher Sci, Hampton, NH, USA). Various assay kits and other molecular biology grade reagents were obtained from various sources, which have been listed along with their respective methods.

### 2.2. Cell Lines and Culture

Human breast cancer cell lines MDA-MB-231 (triple negative breast cancer (TNBC) cells) and MCF-7 (ER positive and PR positive breast cancer cells) were obtained from American Type Culture Collection (ATCC) (Manassas, VA, USA). The cells were grown in Dulbecco’s Modified Eagle’s Medium (DMEM) (Corning Inc., Corning, NY, USA) supplemented with 10% fetal bovine serum (Atlanta Biologicals, Flowery Branch, GA, USA) and 1% penicillin-streptomycin (Corning Inc., Corning, NY, USA), incubated at 37 °C under 5% CO_2_. 3-(4,5-dimethylthiazol-2-yl)-2,5-diphenyltetrazolium bromide (MTT) and crystal violet were procured from Fisher Bioreagents (Fisher Sci, Hampton, NH, USA). Paraformaldehyde (PFA) was procured from Alfa Aesar (Haverhill, MA, USA). Vectashield Hardset mounting medium with DAPI was obtained from Vector Laboratories Inc. (Burlingame, CA, USA).

### 2.3. Preparation of Met Loaded Liposomes

Met loaded liposomes were prepared using a thin-film hydration method. This method was modified [28] by using DPPC:cholesterol in a 7:3 molar ratio for a total of 10 mM with or without DOTAP (5% *w*/*w*). The active and passive loading method was followed as reported previously [28]. Briefly, lipids were dissolved in methanol and dried for 2 h in a round bottom flask under reduced pressure (250 Pa) at 40 °C on a rotary evaporator (WG-EV311, Wilmad Lab-glass, Vineland, NJ, USA). For passive loading (Formulation—Met Pass in Table 1), the dried lipid films were hydrated using an aqueous Met solution of 10 mg/mL for 2 h at 40 °C on a rotary evaporator. The liposomal formulation obtained was bath sonicated for 20 min at 25 °C, followed by extrusion through 200 and 100 nm membrane filters (Nuclepore™, GE Healthcare, Chicago, IL, USA) on a LiposoFast^®^ Extruder device (Avestin Inc., Ottawa, ON, State abbr, Canada) at 65 °C following 21 cycles to obtain small unilamellar vesicles (SUVs). Non-encapsulated drug was separated from Met-loaded SUVs by passing it through a Sephadex-G-25 PD-10 column (equilibrated with PBS pH 7.4). In case of active loading (Formulation—Met pH 3 and Met pH 9 in Table 1), the dried lipid films were hydrated using a 250 mM ammonium acetate solution of varying pH (3.0 and 9.0), and obtained liposomes were extruded as mentioned above. A transmembrane gradient was developed by passing the obtained liposomes through a Sephadex-G-25 PD-10 column by exchanging the outer phase of lipid vesicles with PBS pH 7.4. Ammonium acetate entrapped liposomes were then incubated with drug for 2 h at 65 °C and the free drug was isolated by column separation as described above.

Due to its lower encapsulation efficiency, modification of thin-film hydration method was used as reported previously (Formulation Met ND lipo and Met DO lipo in Table 1) [29]. This method involved addition of Met to lipids together dissolved in methanol and subjected to formation of a film as mentioned above. The film was hydrated using PBS buffer (pH 7.4) for 2 h at 40 °C and further bath sonicated for 20 min at 25 °C. The liposomal suspension was then subjected to size reduction via probe sonication in an ice bath for a total of 3 min (10 s on/off cycle). Liposomes were then centrifuged for 5 min at 3000× *g* to separate the free drug. For the Met DO lipo formulation, DOTAP (5% *w*/*w*) of DPPC concentration was added to make the lipid constituents (Table 1). Blank liposomes were prepared using a similar method but without the addition of Met. All the prepared liposomes were stored at 4 °C for further studies.

### 2.4. Physicochemical Characterization of Liposomes

#### 2.4.1. Particle Size and Polydispersity Index (PDI)

The liposomal size and size distribution were measured by photon correlation spectroscopy using a Zetasizer nano ZS (Malvern Instruments, Malvern, UK). The polydispersity index was evaluated to determine the homogeneity of particle size. The zeta potential was measured by determining the electrophoretic mobility of the liposomes under an electrical field using the Zetasizer nano ZS (Malvern Instruments, Malvern, UK).

#### 2.4.2. Cryo Transmission electron microscopy (TEM)

TEM imaging of liposomes was performed in cryo mode to evaluate the morphology of vesicles. Approximately 10 μL of the sample solution was placed carefully on a lacey carbon-coated copper grids (300 mesh, Ted Pella Inc., Redding, CA, USA). The sample was blotted with a filter paper leaving a very thin film of sample on the grid. The sample was immediately shock-frozen and loaded into a 626-cryo-specimen holder (Gatan, Inc., Pleasanton, CA, USA). The samples were imaged using FEI Titan Halo 80-300 TEM operated at voltage of 100 kV. The images were recorded using 4000 × 4000 Ceta 16M CMOS camera (Gatan, Inc.; Pleasanton, CA, USA) operated under low dose of electron voltage.

#### 2.4.3. UPLC Method of Analysis

The UPLC analysis of Met was achieved using the ACQUITY UPLC™ HSS T3 C18 column (100 mm × 2.1 mm, 1.8 μm). The mobile phase constituted of an isocratic elution of 0.1% orthophosphoric acid and acetonitrile in 50:50 *v/v* ratio at a flow rate of 0.7 mL/min and *λ*_max_ of 237 nm on UPLC (Waters Corp., Milford, MA, USA). The data were collected and analyzed using Empower 3.0 software (Waters Corp., Milford, MA, USA).

#### 2.4.4. Entrapment Efficiency and % Drug Loading

The entrapment efficiency was determined by comparing the amount of drug entrapped in the liposomes to the amount of drug added initially. Briefly, 20 µL of formulation was lysed using a mixture of methanol and acetonitrile and was centrifuged at 20,000 *g* for 45 min to separate the drug from the lysed formulation. The obtained supernatant was subjected to UPLC analysis to determine the amount of drug entrapped.

The % drug loading was evaluated by comparing the amount of drug entrapped in the liposomes to total amount of formulation (amount of lipids + amount of drug).

$$\mathrm{Entrapment}\;\mathrm{efficiency}\%=\left[\frac{\mathrm{Drug}\;\mathrm{entrapped}\;\mathrm{in}\;\mathrm{the}\;\mathrm{liposomes}}{\mathrm{Total}\;\mathrm{drug}\;\mathrm{added}\;\mathrm{initially}}\right]\times 100\;\%\;\mathrm{Drug}\;\mathrm{loading}=\left[\frac{\mathrm{Drug}\;\mathrm{entrapped}\;\mathrm{in}\;\mathrm{the}\;\mathrm{liposomes}}{\mathrm{Total}\;\mathrm{lipids}\;\mathrm{added}}\right]\times 100$$

#### 2.4.5. In Vitro Drug Release

In Vitro drug release studies from the liposomal formulations were carried out using dialysis cassettes (2000 MWCO, 0.1–0.5 mL, Thermo Scientific, Waltham, MA, USA). Briefly, the cassettes were hydrated previously in PBS (pH 7.4) and 300 µL of formulation was loaded in the cassette using a syringe. The cassettes were immersed in 150 mL of release media (1% tween-80 dissolved in PBS (pH 7.4) and were incubated at 37 ± 1 °C with stirring at 100 rpm. Samples were withdrawn at specific time intervals and release media was replenished with equal volume of fresh media maintained at 37 ± 1 °C. The samples were analyzed using UPLC method as described previously in Section 2.4.3.

### 2.5. Differential Scanning Calorimetry (DSC) Studies

The differential scanning calorimetry was performed using DSC 6000 (PerkinElmer, Waltham, MA, USA) connected with intra-cooler accessory. Approximately 3 mg of sample was sealed in aluminum pan and analyzed over a temperature range of 30 °C to 300 °C against an empty pan maintained as reference using the closed pan technique. The heating rate was 10 °C/min under a nitrogen purge maintained at a flow rate of 50 mL/min.

### 2.6. Powder X-ray Diffraction (PXRD) Studies

The diffractometry was performed on an XRD-6000 (Shimadzu, Kyoto, Japan) using a graphite monochromator and copper source with Kα radiation (*λ* of 1.5418 Å) obtained at a voltage of 40 kV and a current of 30 mA. The samples were analyzed after spreading as a thin film on a micro glass slide to obtain uniform spreading of the sample. Analysis was performed over 10° to 80° 2*θ* range at a scanning rate of 2° 2*θ*/min.

### 2.7. Stability Studies

The stability of optimized liposomal formulations was evaluated over a period of 4 weeks. Briefly, the formulations were stored at 4 °C, 25 °C (room temperature), and 37 °C; and samples were withdrawn at specific time intervals. The samples were analyzed for size, zeta potential, and amount of drug entrapped.

The formulations were also exposed to 40 ± 1 °C and 75 ± 2% relative humidity (RH) and were analyzed using DSC and PXRD to study effect of accelerated stability conditions.

### 2.8. In Vitro Cellular Uptake Studies

Cellular uptake of the liposomes was assessed using coumarin-loaded liposomes prepared using the same method by replacing Met with coumarin. For intracellular uptake, MCF-7 and MDA-MB-231 cells were seeded in 8-well chamber slides (Eppendorf, Hauppauge, NY, USA) at a density of 1.0 × 10^4^ cells/chamber and were incubated overnight at 37 °C with 5% CO_2_. The cells were incubated with either plain coumarin or coumarin-loaded formulations at a concentration of 1 µg/mL for 3 h. After incubation, the cells were washed thrice with ice-cold PBS (pH 7.4) and fixed using 4% paraformaldehyde. The cells were again washed twice with PBS; the coverslip was removed from the chamber and mounted with VECTASHEILD hardset mountant containing DAPI (H1500, Vector laboratories, Burlingame, CA, USA). The mounting media was allowed to harden overnight at 4 °C, and slides were then imaged using a fluorescence microscope. (EVOS-FL, Thermo Fisher Scientific, Waltham, MA, USA).

### 2.9. In Vitro Cytotoxicity Assay

Cytotoxicity of the formulations were evaluated using an MTT assay as described previously [30]. The IC_50_ of the formulations were evaluated in the cell lines mentioned above and were compared to Met. Briefly, human breast cancer cell lines MDA-MB-231 and MCF-7 were seeded in 96-well plates with a density of 2.5 × 10^3^ cells/well and incubated overnight at 37 °C with 5% CO_2_ to allow surface attachment. Various concentrations of Met-loaded liposomal formulations were prepared by diluting liposomal stock with media. The cells were treated with different concentrations of plain Met and Met-loaded liposomes, and then incubated for 72 h. Subsequent to 72 h treatment, media was removed and MTT (1 mg/mL) was added, followed by 2 h of incubation. The formazan crystals formed during incubation were dissolved in DMSO after aspirating the MTT solution. Plates were shaken for 30 min on plate shaker and the optical absorption was measured at 570 nm using a TECAN plate reader (Tecan Group Ltd., Männedorf, Switzerland). The cytotoxic potential of treatment groups (*n* = 6 per group, for 3 individual experiments) were determined by comparing cell viability against the non-treated cells used as control groups. The cytotoxicity data were transformed and normalized for calculation of IC_50_. IC_50_ values were calculated graphically by non-linear curve fitting module in Graph Pad prism 6.01 software (San Jose, CA, USA).

Cytotoxicity of blank DOTAP liposomes was evaluated on LLC-PK1 cells, porcine kidney tubular epithelial cells; HEK-293, human embryonic kidney cells; and MCF-7, breast cancer cells by following the same protocol.

### 2.10. Cell Migration Assay

The cell migration ability of cells was assessed using the wound healing assay as described previously [31]. Briefly, MDA-MB-231 and MCF-7 cells were seeded in a 24-well plate at a density of 2.0 × 10^5^ cells/well and were incubated overnight to adhere. After incubation, a scratch was made in the cell monolayer using a sterile 200 µL micropipette tip. The scratched cells were removed by washing twice with sterile PBS (pH 7.4) and then incubated with different concentrations of Met and Met-loaded liposomes for 24 h. (*n* = 3) time period. The scratch was imaged using an inverted microscope (LAXCO, Bothell, WA, USA) and every image was examined and captured at the same location. The first image of the scratch captured before treatment was considered as time zero. The migration areas were observed as the gap created by the scratch, and the migration was calculated by measuring the distance (gap) between the two edges of scratch at each time interval using ImageJ software (Version 1.44).

### 2.11. Clonogenic Assay

To determine in vitro cell survival after treatment, clonogenic assay was performed as reported previously [32,33]. Both cell lines, MCF-7 and MDA-MB-231, were plated in a 6-well plate at a seeding density of 2.5 × 10^2^ cells per well and incubated overnight at 37 °C (5% CO_2_). The cells were then treated with varying concentrations of Met and Met-loaded liposomes for 48 h, followed by its removal and addition of fresh media. The cells were further cultured for 7 days by replacing the media every 2 days. After the incubation period, colonies were washed twice with ice-cold PBS (pH 7.4) and fixed using 4% paraformaldehyde for 10 min. The colonies were incubated with 0.2% crystal violet, followed by three rinses with tap water to remove excess dye. After air drying, the colony numbers were counted using OpenCFU colony counter software (Version 3.8) and the images were captured using a digital camera.

### 2.12. Caspase Enzymatic Activity Assay—Apoptosis-Induced Cell Death

Caspase enzymatic activity assay was performed to determine the apoptotic effect of Met and Met-loaded liposomes. The assay was performed using an EnzChek^®^ caspase-3 assay kit (Molecular Probes, Eugene, OR, USA) as previously reported [34,35]. This assay kit is based upon the fluorometric detection of a bright blue fluorescent product obtained by proteolytic cleavage of 7-amino-4-methylcoumarin-derived substrate Z-DEVD-AMC. Briefly, 1.0 × 10^6^ cells/plate were seeded in petri dishes followed by treatment for 6 h with Met, blank DO lipo, and Met DO lipo along with a control group maintained. After treatment, cells were washed twice with ice-cold PBS (pH 7.4) and were scraped to obtain a cell suspension. The obtained cell suspensions were centrifuged to get cell pellets, which were then lysed using lysis buffer. The lysed pellets were centrifuged to separate the cell debris and the supernatant obtained was transferred to 96-well plate. Equal volume of substrate was added to the supernatant and the fluorescence was measured at excitation/emission 342/441 nm. Control without enzyme was used to estimate the background florescence of substrate in each assay.

### 2.13. Western Blot Analysis

MDA-MB-231 cells were seeded in petri dish at a density of 1.0 × 10^6^ cells/plate and incubated overnight. The following day, cells were treated with Met and Met DO lipo formulations and incubated for 72 h. After treatment, the cells were scraped to collect the pellets, washed 3 times with PBS, and subjected to lysis using 1% Triton^®^ X-100 (BP151-500, Fisher Bio-Reagents, Hampton, NH, USA) and 1% Halt™ Protease and Phosphatase Inhibitor Cocktail (PI78441, Thermo-Fisher Scientific, Waltham, MA, USA) in PBS followed by sonication for 90 min at 4 °C. Samples were centrifuged for 15 min at 4 °C at 13,000 g and the supernatants were collected. Cell lysate protein was quantified by the DC™ Protein Assay Kit (Bio-Rad, Hercules, CA, USA). The samples were mixed with 2× Laemmli buffer (#161-0737, Bio-Rad) and 2-mercaptoethanol and denatured at 110 °C for 10 min.

For Western blot analysis, 10 µg of protein was loaded and separated on 4–20% Mini-PROTEAN^®^ TGX™ Precast Protein Gels (Bio-Rad) using a Bio-Rad PowerPac^TM^ Basic Power Supply and transferred to Trans-Blot^®^ Turbo™ Midi PVDF membranes (Bio-Rad) using a Bio-Rad Trans-Blot^TM^ Turbo Transfer System.

The membranes were blocked with 5% bovine serum albumin in PBS and probed with corresponding primary antibodies (1:1000 dilution) overnight at 4 °C. The following antibodies were used: β-actin (4970S, Cell Signaling Technology, Beverly, MA, USA); p-mTOR (sc-293133, Santa Cruz Biotechnology, Dallas, TX, USA); and p-AMPK (50081S, Cell Signaling Technology, Beverly, MA, USA). Membranes were then incubated with the corresponding secondary HRP-conjugated antibodies ab6789 (1:10,000 dilution) and ab6721 (1:10,000 dilution) (Abcam, Cambridge, UK) for 1 h at room temperature and were subjected to Western Bright chemiluminescence (WBF25, Gel Company Inc., San Francisco, CA, USA). Protein bands on the membranes were visualized using the Chemi mode by the Omega Lum™ G Imaging System (Gel Company Inc., San Francisco, CA, USA). The band intensity was quantified using ImageJ software (Version 1.44).

### 2.14. In Vitro Tumor Simulation Studies

It is well established that the tumor microenvironment plays an important role in its progression and development [36]. This can be explored by developing 3-D spheroids, which would simulate the microenvironmental tumor conditions and thus serve as a significant tool for improving relevance to in vitro results [37]. The cells were seeded in 96-well plate Nunclon Sphera™ (U bottom) (Thermo-Fisher Scientific, Waltham, MA, USA) at density of 5.0 × 10^2^ cells/well and were incubated overnight at 37 °C with 5% CO_2_. The cells were then subjected to two individual treatment regimens; (i) single dose and (ii) multiple dose. Briefly, single-dose treatment involved treating the cells only once and the media was replaced with treatment free-media every 3 days until 15 days. In the case of multiple doses, repeated drug exposure was performed every 3 days for 15 days. In both the doses, the replacement was done only for half of the media volume to avoid any disturbance to the developed spheroid or evade any chance of aspirating spheroid during complete media removal. The spheroids (*n* = 6, per treatment group) were imaged at specified time intervals using inverted microscope (LAXCO, Bothell, WA, USA). The volume of spheroids was calculated using ImageJ software (Version 1.44).

### 2.15. Statistical Analysis

The results are presented as mean ± SD (unless otherwise stated). Statistical analyses were performed with GraphPad Prism 6.01 (San Jose, CA, USA) using Student’s *t*-test, one-way ANOVA and Tukey’s post-hoc multiple comparison. Statistical significance was considered at *p* < 0.05.

## 3. Results

### 3.1. Formulation Fabrication, Optimization, and Characterization

**Figure 1:**
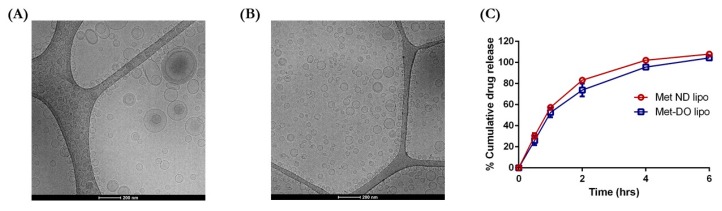
Preliminary characterization of liposomal formulations during development and optimization. (**A**) Cryo TEM micrographs of metformin hydrochloride (Met) ND lipo. Scale bar = 200 nm. (**B**) Cryo TEM images of Met DO lipo. Scale bar = 200 nm. (**C**) In Vitro drug release profile of optimized formulation, Met ND lipo, and Met DO lipo as revealing all the drug being released with 6 h. Data represent mean ± SD (*n* = 3).

The liposomes were formulated using various techniques as shown in Table 1. Met exhibited a low entrapment (5.2 ± 0.2%) using a passive loading method with an average particle size of 136.3 nm and narrow size distribution. To enhance drug entrapment, we switched to an active loading method using an ammonium sulfate-based transmembrane gradient established with varying pH. The pH used for maintaining the gradient during active loading was pH 3.0 and 9.0. As compared to passive loading, % entrapment of Met increased to 9.9 ± 1.6% with the transmembrane gradient method at pH 9.0. The entrapment was further increased by ~2.5-fold by decreasing the pH of the rehydrating medium to pH 3.0 (24.9 ± 3.9%). The data indicate entrapment of the drug to be influenced by the pH of the hydrating medium. In the transmembrane gradient method, a pH gradient is established between the internal and external environment of liposomes. Therefore, upon entering liposomes, drug molecules will get protonated/ionized due to a pH variation and the ionized species will not be able to cross the lipophilic barrier and will be retained in liposomal vesicles [28,38]. Met, a strong base (pKa~12.4), will exist largely as hydrophilic cationic species at pH 3.0 as compared to pH 9.0. Due to the liposomal protonation, the ionized species will remain encapsulated resulting in higher drug entrapment. However, there was no significant difference in particle size with 129.6 ± 10.1 nm for Met pH 3.0 and 136.3 ± 4.1 nm for Met pH 9.0 (Table 1). The surface charge value though displayed an increase from −2.6 ± 0.8 mV at pH 3.0 to 0.6 ± 0.3 mV at pH 9.0 with PDI values about 0.25 for both formulations (Table 1). Even though the active loading method resulted in increased % entrapment of drug, we wanted to load more drug. To this end, we used a modified passive loading method by adding Met along with lipid components to form a drug-loaded dried lipid film. This modification resulted in a ~11.2-fold increase in entrapment as compared to passive loading, which led to 58.2 ± 4.5% entrapment of Met. During passive loading, addition of Met to hydration medium (i.e., PBS buffer, pH 7.4) caused ionization of most of the drug molecules. Once ionized, Met is not able to penetrate through the lipid membrane and cannot be entrapped in liposomal vesicles. However, in the modified passive loading method or drug-loaded film method, Met is incorporated in the lipid layer during film formation process. Upon hydration of drug loaded lipid film with buffer, ionization of Met would not impact entrapment as molecules of Met are already embedded in a lipid bilayer matrix and will not have to struggle entering in the vesicles. This will result in increased entrapment of Met molecules in liposomes. The particle size was reduced to 102.3 ± 1.12 nm with a surface charge of −1.2 ± 0.9 mV as seen for Met ND lipo (Table 1). Incorporation of cationic lipid, DOTAP, in Met DO lipo formulation using drug loaded film method produced vesicles of 98.2 ± 2.9 nm size with increased % drug entrapment to 65.6 ± 5.2% (Table 1). In terms of % drug loading, Met ND lipo displayed 19.4 ± 1.5%, resulting in ~69 mM Met concentration, whereas Met DO lipo possessed 21.9 ± 1.7% drug loading, accounting for almost 79 mM Met concentration loaded in liposomes (Table 1). The presence of a cationic lipid in the delivery system intensified zeta potential value of ~40 mV (Table 1). As seen in Figure 1A, cryo TEM micrographs of Met ND lipo demonstrates the presence of spherical vesicles with particle size in accordance to the results of photon correlation spectroscopy. The surface morphology of Met DO lipo as observed in Figure 1B also displays spherical liposomal vesicles with particle size ~100 nm. Based on the data obtained, Met ND lipo and Met DO lipo were optimized formulations complying with required particle size and higher % entrapment efficiency and were selected for further studies.

### 3.2. In Vitro Drug Release

In Vitro release of drug from optimized liposomal formulations (i.e., Met ND lipo and Met DO lipo) was evaluated in PBS, pH 7.4, along with 1% Tween-80 at 37 °C. The data in Figure 1C demonstrate initial fast release of 30.4 ± 2.6% and 26.1 ± 4.5% for Met ND lipo and Met DO lipo, respectively, observed for 0.5 h. The release later reached ~50% after 1 h followed by an increase in release of 83.7 ± 1.0% for Met ND lipo and 73.8 ± 6.1% for Met Do lipo, as observed at 2 h. This was followed by a slower release continued for up to 6 h for complete release to take place. The increased release can be due to the hydrophilic nature of Met in addition to its low molecular weight, which may aid in the easy release of the drug from the liposomal matrix [39]. Moreover, a lower glass transition (*T*_m_) temperature of DPPC (~39–41 °C) [40], which is similar to a physiological temperature of 37 °C (also used for release studies), may cause disruption of liposomes, thereby allowing enhanced drug release. However, drug release from Met DO lipo was comparatively less than Met ND lipo, which can be accredited to the fact that addition of DOTAP causes stringent molecular arrangement of the lipid bilayer resulting in slower release of the drug [41].

The release data were further subjected to mathematical modeling using zero-order, first-order, Higuchi, and Korsmeyer-Peppas models to understand the release kinetics [42]. In order to understand the release mechanism, a release equation was determined from every model for fitting. The optimized equation was then chosen based on the value of correlation coefficient (*r*^2^). As can be seen from results in Table 2, the release profile of Met from both liposomal formulations best fitted the first-order release mechanism, owing to highest value of r^2^. Thus, the in vitro release of Met from liposomal vesicles can be described by first-order release kinetics indicating a concentration dependent release. While the in vivo environment cannot be completely mimicked by in vitro studies, the in vitro drug release only provides an insight. Further studies are required to evaluate the in vivo release of Met from liposomal delivery system.

### 3.3. Thermal Analysis (DSC Studies)

**Figure 2:**
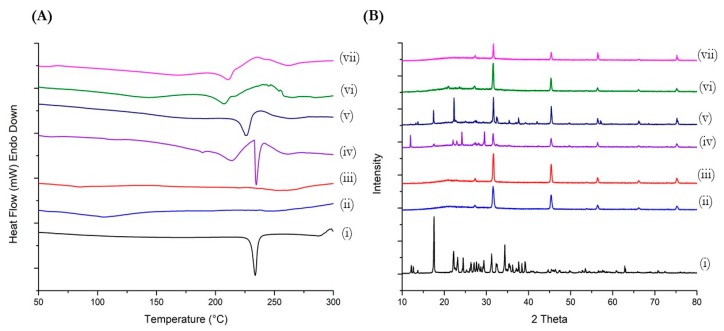
Characterization of optimized liposomal formulations performed using (**A**) Differential scanning calorimetry (DSC) and (**B**) Powder X-ray diffraction (PXRD) to confirm encapsulation of drug in liposomal vesicles. (i) Met hydrochloride; (ii) blank ND lipo; (iii) blank DO lipo; (iv) physical mixture of blank ND lipo and Met; (v) physical mixture of blank DO lipo and Met; (vi) Met ND lipo; and (vii) Met DO lipo.

The calorimetric data obtained from DSC reveal a sharp endothermic peak representing the melting point of Met at 234 °C (Figure 2A). This characteristic Met peak is absent in thermograms of both blank liposomes and Met-loaded liposomes. However, physical mixtures of blank liposomes/blank DOTAP liposomes and Met displays sharp endothermic peak for Met at 234 °C and 228.2 °C, respectively. These results indicated the successful encapsulation of Met in liposomal vesicles.

### 3.4. Powder X-ray Diffraction (PXRD) Studies

Diffractometry studies revealed characteristic crystalline peaks of Met over 10° to 30° 2*θ* range as can be seen in Figure 2B. The characteristic peaks of Met were observed at 12.15°, 18.2°, 22.2°, 24.5°, 29.5°, and 32.3° 2*θ* values. The analysis of blank ND lipo and blank DO lipo also shows a few characteristic peaks of the liposomal formulation at a range of 25°–32° 2*θ* values, specifically at 27.25° and 31.7° 2*θ* values, which coincides with peaks of Met. In addition, other peaks for both blank formulations are found to be at 45.32°, 56.5°, 66.2°, and 75.2° 2*θ* values. The diffractogram of physical mixtures comprising of blank ND lipo/blank DO lipo and Met shows presence of Met characteristic peaks along over range of 12°–40° 2*θ* values and blank liposomal peaks in 30°–80° 2*θ* values, thus confirming the presence of both liposomal formulation as well as Met molecules. Upon analyzing Met-loaded liposomal formulations (i.e., Met ND lipo and Met DO lipo), Met characteristic peaks were absent over the entire range of 10°–40° 2*θ* values, however, the liposomal characteristic peaks were consistently found in both diffractograms at their respective 2*θ* values (Figure 2B). The results obtained from PXRD-studies indicate successful encapsulation of Met in liposomal vesicles.

### 3.5. Stability Studies

**Figure 3:**
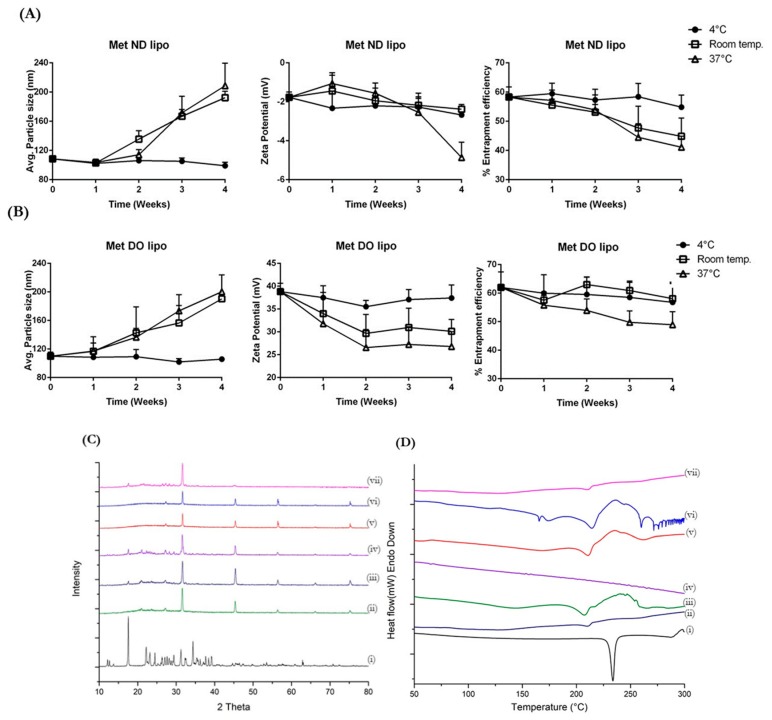
Stability of liposomal formulations evaluated at different temperatures; 4 °C, room temperature (~25 °C), and 37 °C, expressed as mean particle size, zeta potential, and % entrapment efficiency. (**A**) Stability of Met ND lipo and (**B**) Met DO lipo evaluated for various parameters over period of four weeks. (**C**) Accelerated stability studies of liposomal formulations performed at 40 °C/75% RH for 45 days analyzed using PXRD and (**D**) DSC. (i) Met hydrochloride; (ii) Met ND lipo at 4 °C; (iii) Met ND lipo at Room temperature; (iv) Met ND lipo at 37 °C; (v) Met DO lipo at 4 °C; (vi) Met DO lipo at Room temperature; and (vii) Met DO lipo at 37 °C. Data represent mean ± SD (*n* = 3).

Lipid-based formulations are known to suffer several stability issues. Therefore, development of a stable liposomal formulation is a necessity for designing a successful delivery system. The stability of optimized liposomal formulations—Met ND lipo and Met DO lipo—was evaluated in terms of average particle size, surface charge and % drug entrapment as a function of temperature, and storage period. In addition, to understand the impact of accelerated stability storage conditions upon liposomes, formulations were also exposed to 40 °C/75% RH conditions and analyzed using DSC and PXRD studies. The entrapment efficiency of Met ND lipo was found to be stable at 4 °C with ~5–7% drug entrapment reduction after four weeks, as seen in Figure 3A. There was minimal impact on the particle size and surface charge of liposomes, which were found to be 108.4 ± 4.9 nm and −1.7 ± 0.1 mV, respectively, after 28 days of storage at 4 °C. However, storing at room temperature (~25 °C) and 37 °C resulted in 13–20% reduction in % drug entrapment along with increased particle size of ~210 nm. The surface charge after four weeks of storage displayed a reduction in zeta potential values, which were found to be −2.4 ± 0.3 mV and −4.9 ± 0.8 mV at 25 °C and 37 °C, respectively (Figure 3A). As shown in Figure 3B, the storage of Met DO lipo at 4 °C and 25 °C resulted in < 5% reduction in % drug entrapment after 28 days of storage. The particle size and zeta potential at 4 °C was reported to be 99.1 ± 3.4 nm and 37.4 ± 2.8 mV, respectively, after storing for four weeks, indicating adequate dispersion of vesicles. However, storage at 25 °C for four weeks resulted in aggregation of particles leading to a 190.3 ± 6.1 nm particle size, which in turn impacted surface charge causing reduction of zeta potential to 30.13 ± 2.6 mV. At a physiological temperature of 37 °C, % drug entrapment displayed a ~15% reduction after 15 days of storage, which is about a 3-fold reduction as compared to 4 °C and 25 °C. The particle size at 37 °C was found to be increased up to 200.3 ± 23.5 nm along with reduced zeta potential value of 26.8 ± 3.2 mV (Figure 3B).

Accelerated stability studies were conducted on Met ND lipo and Met DO lipo formulations stored at 40 °C/75% RH for period of 45 days. As shown in Figure 3C, PXRD data indicate the presence of Met characteristic peaks in Met ND lipo and Met DO lipo over range of 15°–30° 2*θ* values. These peaks were absent in diffractograms of formulations stored at 4 °C and 25 °C, which indicates leaching of encapsulated drug from vesicles to the surface of liposomes at a higher temperature (~40 °C). Additionally, there is loss of liposomal peaks as reported by diminishing of peaks at 56.5°, 66.2°, and 75.2° 2*θ* values while these peaks are present in both the formulations exposed to 4 °C and 25 °C for 45 days. Similarly, upon analyzing DSC data of accelerated study (Figure 3D), it can be observed that a broad endothermic peak at 220 °C found in Met ND lipo and Met DO lipo stored at 4 °C and 25 °C was diminished in both formulations when exposed to accelerated conditions of 40 °C/75% RH indicating influence on liposomal integrity. Interestingly, no endothermic peak of the Met melting point was observed in any of the formulations stored at accelerated conditions. The changes in formulations detected at higher temperatures or accelerated conditions can be due to exposure of liposomal vesicles at temperatures closer to the glass transition temperature (*T*_m_) of DPPC (~41 °C) resulting in distorted integrity of liposomes and subsequent leaching out of drug from the vesicles. The in vitro stability studies performed on optimized liposomal formulations indicate that both formulations were found to be stable at 4 °C (storage temperature) as evaluated for a period of 45 days while stable at room temperature (~24 °C) for two weeks.

### 3.6. Cellular Uptake Studies

**Figure 4:**
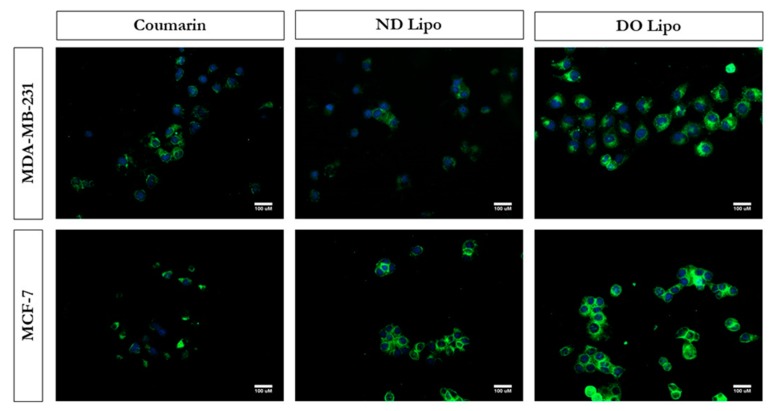
In Vitro cellular uptake images of coumarin-loaded liposomes and coumarin solution (used as control) determined using florescence microscopy in MDA-MB-231 and MCF-7 breast cancer cells after 3 h of incubation. Representative images from *n* = 3 experiments. Scale bar = 100 µm.

Liposomal-mediated drug delivery has been shown to enhance intracellular delivery, and this method has been widely exploited in many anti-cancer treatments. The inclusion of cationic lipids (DOTAP) in liposomes is known to escalate the cellular uptake of liposomal vesicles compared to neutral or negatively charged liposomes [43]. This enhancement in cellular internalization can be attributed to the fact that DO lipo possess a positive surface charge on liposomal vesicles, which would allow liposomes to remain in closer vicinity to the cells (charged negatively due to phospholipids). This will aid in increasing the probability of being internalized by the cells. In Vitro cellular uptake studies were performed to evaluate uptake efficiency of positively charged versus neutral liposomes in breast cancer cells. The study was performed using coumarin, a fluorescent dye, coumarin-loaded ND lipo, and coumarin-loaded DO lipo formulations monitored using florescence microscopy. Figure 4 represents florescent images of cellular uptake studies in MDA-MB-231 and MCF-7 cell lines.

The results show an increase in florescence due to enhanced cellular uptake of liposomal formulations as compared to coumarin. Amongst both liposomal formulations, DO lipo displayed a greater increase in florescence intensity in both cell lines as compared to ND lipo. Thus, it was shown that DO lipo possess a greater internalization potential in breast cancer cells.

### 3.7. Cytotoxicity Studies

**Figure 5:**
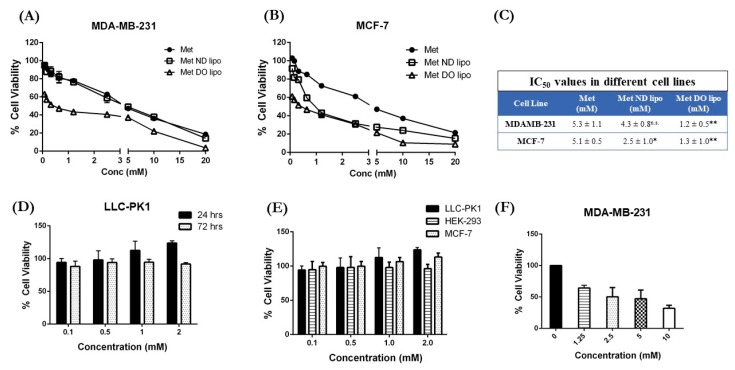
Cytotoxicity studies of Met-loaded liposomes determined using MTT assay after 72 h incubation with treatment in two different breast cancer cell lines: (**A**) MDA-MB-231 and (**B**) MCF-7; (**C**) IC_50_ values for each treatment in both cell lines; (**D**) Blank 1,2-dioleoyl-3-trimethylammonium-propane (DOTAP) liposomes cytotoxicity evaluated on LLC-PK1 cell lines; (**E**) % Cell viability of blank DOTAP liposomes evaluated after 24 h of incubation in LLC-PK1, HEK-293, and MCF-7 cells; (**F**) Met Do lipo cytotoxicity in MDA-MB-231 after 6 h of incubation. Data represent mean ± SD of three individual experiments with *n* = 6 for each trial. * *p* < 0.05 and ** *p* < 0.01 vs. Met.

The cytotoxic potential of developed liposomal formulations was evaluated in two breast cancer cells: MDA-MB-231 and MCF-7, using MTT assay. As can be seen in Figure 5, Met exhibits an IC_50_ value of 5.3 ± 1.1 mM in MDA-MB-231 cells, which was reduced to 4.3 ± 0.8 mM by Met ND lipo. However, Met DO lipo produced a 4.5-fold reduction in IC_50_ value as compared to Met or Met ND lipo. The IC_50_ value for Met DO lipo was found to be 1.2 ± 0.5 mM (*p* < 0.01 against Met and Met ND lipo) in MDA-MB-231 cells, demonstrating enhanced anti-proliferative efficacy of Met upon encapsulation in DO lipo as compared to Met or Met ND lipo (Figure 5A).

Investigation of the in vitro cytotoxic effect against MCF-7 cells, as shown in Figure 5B, also showcase a promising effect of Met encapsulated in cationic liposomal formulation against Met. About ~2-fold reduction in IC_50_ value was observed with Met ND lipo treatment with IC_50_ values of 2.5 ± 1.0 mM (*p* < 0.05) as compared to 5.1 ± 0.5 mM for plain Met (Figure 5C). This reduction was significantly (*p* < 0.01) increased by ~3.9-fold via treatment of Met DO lipo, as the IC_50_ value decreased to 1.3 ± 1.0 mM. The reduction in IC_50_ value of Met DO lipo in both cell lines can be supported by the hypothesis that enhanced intracellular localization in cells, in agreement with cellular uptake studies, leads to increased cytotoxic potential. The obtained cytotoxicity results signify achievement of the study’s primary objective, which was to reduce the dose required for Met to exhibit anti-tumor activity in breast cancer cells.

Furthermore, evaluation of blank DO liposomes on normal kidney cells, LLC-PK1, as shown in Figure 5D, reveals that the DO liposomes demonstrate ~100% cell viability at 24 h and ~90% cell viability at 72 h. Similarly, cytotoxicity in HEK after 24 h was found to be ~90% (Figure 5E), while the blank Do lipo displayed ~100% cell viability after 24 h incubation in MCF-7 cells as seen in Figure 5E. Additionally, incubation of Met DO lipo in MDA-MB-231 cells for 6 h demonstrated 50% cell viability at ~2.5 mM. This indicates safety and tolerability of DOTAP liposomes in normal tissues, when used at and above IC_50_ concentrations.

### 3.8. Cell Migration Assay

**Figure 6:**
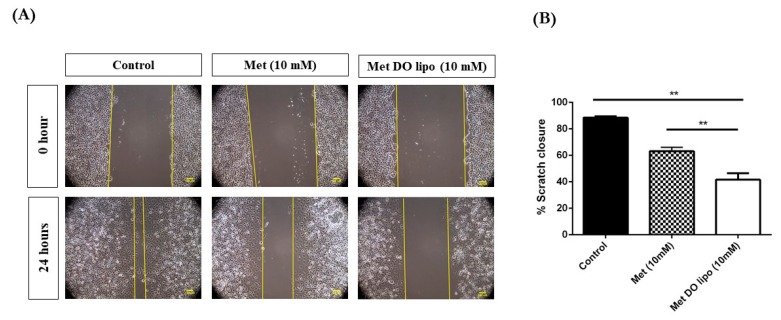
Effect of treatment on cell migration property of MDA-MB-231. (**A**) Microscopic images of scratch after different incubation periods with treatment: 0 h and 24 h. Scale bar = 100 µm (**B**) Quantitative representation of treatment effect on cell migration of MDA-MB-231 cells treated with Met and Met DO lipo expressed as % scratch closure at various time intervals. Data represent mean ± SD (*n* = 3). ** *p* < 0.01.

The migration of cells is an important step in initiating metastasis; a pivotal nature of cancerous cells forming solid tumors [44]. In order to study the effects of Met encapsulated liposomal formulation on the cell migration and metastatic activity of cells, a scratch-based cell migration assay was performed on MDA-MB-231 breast cancer cells. Figure 6A represents pictures of this scratch assay where a sterile microtip was used to create a gap (scratch) in the cells, which then underwent 0 and 24 h treatment with control (media), Met (10 mM), or Met DO lipo (10 mM) The concentration 10 mM was chosen based on the reported IC_50_ value (>20 mM) of Met in MDA-MB-231 after 24 h of treatment [45,46,47]. After 24 h of incubation, significant cell migration was observed in control groups (no treatment) with 88.5 ± 1.4% scratch closure. However, this migration effect was significantly inhibited in treatment groups of Met (63.1 ± 3.0% scratch closure) and Met DO lipo (41.7 ± 4.9% scratch closure); amongst which inhibition of the scratch closure was found to be significant (*p* < 0.01) (Figure 6B). Based on these results, it can be concluded that liposomal encapsulation of Met causes enhanced inhibition of breast cancer cell migration when compared to Met, which in turn could help to prevent tumor metastasis.

### 3.9. Clonogenic Assay

**Figure 7:**
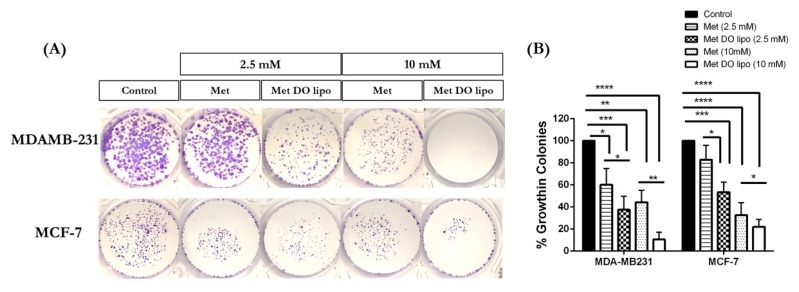
Effect of treatment on colony formation in MDA-MB-231 and MCF-7 cell lines after 24 h of incubation, followed by incubation in fresh media for additional seven days. (**A**) Images of colonies stained with crystal violet. (**B**) Quantification of treatment effect on clonogenic properties of MDA-MB-231 and MCF-7 cells treated with Met and Met DO lipo expressed as % growth in colonies at different dose; 2.5mM and 10mM. Data represent mean ± SD (*n* = 3). * *p* < 0.05, ** *p* < 0.01, *** *p* < 0.001 and **** *p* < 0.0001.

The clonogenic assay is an in vitro tool used to evaluate the ability of a single cell to develop into a colony of cells (≥ 50 cells) by proliferating indefinitely. This is also known as clonal expansion [48,49]. It is useful to evaluate the effect of various chemotherapeutic agents on cell survival and proliferation by determining the fraction of the cells possessing reproductive integrity after treatment and which may have the potential to develop as tumor [49]. Figure 7A exemplifies that treatment with Met DO lipo (2.5 mM) against MDA-MB-231 cells exhibited a significant response with 37.5 ± 10.5% of colonies present as compared to the control at 2.5 mM, and 10.6 ± 6.5% of control present at 10 mM, against considering the control (drug-free treatment) to be 100% (Figure 7B).

MCF-7 cells result in a significant (*p* < 0.01) reduction in the number of colonies (53.5 ± 9.1% of control) as compared to Met (82.8 ± 13.0% of control); with control treated cells being 100% colonies (Figure 7A,B). The dose increase to 10 mM resulted in decreased colony formation with Met DO lipo (21.6 ± 6.8% of control), with the control being considered as 100% (Figure 7B).

Therefore, it can be stated that encapsulation of Met in liposomes results in a significant reduction in the number of colonies, which may further inhibit the stem-ness of breast cancer cells, and therefore will potentially inhibit the metastasis of breast cancer.

### 3.10. Caspase-3 Enzymatic Activity: Evaluating Apoptosis-Induced Cell Death

**Figure 8:**
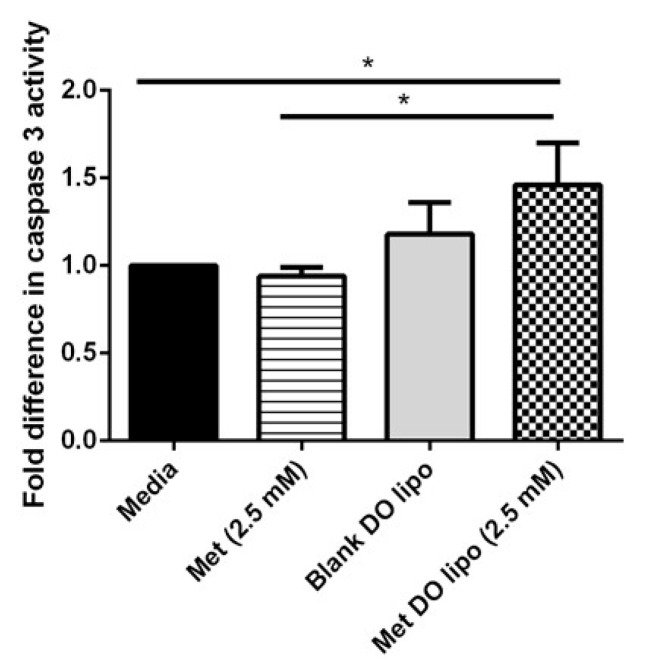
Effect of treatment on caspase-3 activity in MDA-MB-231 cells determined using EnzChek^®^ caspase-3 assay kit. A significant difference (*p* < 0.05) is observed as compared to control and plain Met when treated with liposomal encapsulated met. Blank (drug-free) liposomal formulation did not show any significant difference compared to any of the treatment groups or control. Data represent mean ± SD (*n* = 3). * *p* < 0.05.

Caspase 3 is an executioner molecule for inducing apoptosis in cells by causing down-regulation of mitochondrial activation resulting in DNA fragmentation and chromatin condensation [50]. Previous studies have demonstrated that Met induces apoptosis in breast cancer cells through a caspase-dependent pathway [51,52,53]. To determine the apoptotic mechanism of cell death instigated by Met and liposomal Met treatment, caspase-3 enzymatic activity was determined using an EnzChek^®^ caspase-3 assay kit (Molecular Probes, Eugene, OR, USA). The increase in amount of caspase-3 activity has a linear relationship with apoptotic activity. Figure 8 shows a significant (*p* < 0.05) increase in caspase-3 enzymatic activity with Met DO lipo treatment as compared to Met or the control. With Met DO lipo (2.5 mM), a 1.5 ± 0.2-fold difference was observed as compared to the control, which is significantly higher than both plain Met and blank DO lipo (no observed difference). All comparisons were made as relative to the media control, which was normalized to 1.0 (Figure 8). These results signify that caspase-3 cascade may be triggered with liposome encapsulated Met treatment, which induces cell death in breast cancer cells.

### 3.11. Western Blot—Understanding the Mechanism of Tumor Growth Inhibition

Western blot analysis was performed to understand the underlying molecular mechanisms by which Met encapsulated liposomes inhibits tumor growth in breast cancer cells. Met is known to modulate the AMPK/m-TOR pathway resulting in anti-tumor activity. AMPK plays a key role in cellular energy metabolism along with maintaining several signaling pathways. The activation of AMPK causes subsequent inhibition of m-TOR, thereby impacting protein synthesis and cellular proliferation, ultimately resulting in the inhibition of tumor growth. Met induces AMPK and downregulation of m-TOR in breast cancer cells [32,54]. The effect of Met encapsulated liposomes was assessed to understand its role in these pathways.

**Figure 9:**
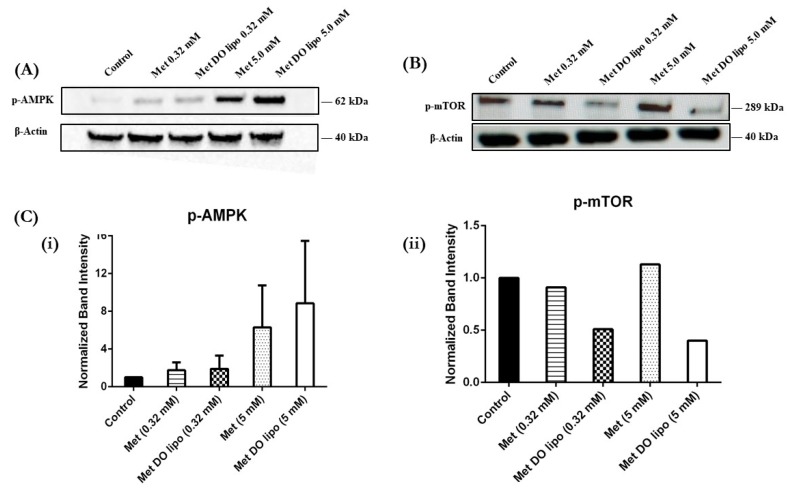
Effect of treatment on cellular markers representing anti-tumor activity. (**A**) Representative Western blots to understand the effect of treatments on p-AMPK activity after 24 h of treatment at varied concentration. (**B**) Representative Western blots to understand the effect of treatments on p-mTOR activity after 24 h of treatment at varied concentration. (**C**) Representative quantified analysis of p-AMPK and p-mTOR (normalized) band intensity determined using ImageJ. As can be seen, Met DO lipo displays increased activation of p-AMPK (increased band intensity) and inhibition of p-mTOR (diminished band intensity) as compared to Met. The effect was seen to be concentration dependent. Representative images from *n* = 1 (for p-mTOR) to two (for p-AMPK) experiments.

As shown in Figure 9A,C(i), treatment with Met DO lipo (5 mM) increased AMPK phosphorylation in MDA-MB-231 cells, as compared to Met, as evident by the increased band intensity corresponding to p-AMPK. Figure 9B,C(ii) demonstrates the effect of treatment on p-mTOR activity, wherein Met DO lipo markedly downregulated p-mTOR as compared to Met at both 0.32 mM and 5 mM. The diminished band intensity is evident in Figure 9A. Upon quantification of the band intensity, it was determined that Met (0.32 mM) showed no effect, whereas Met DO lipo (0.32 mM) displayed a 2-fold reduction in band intensity (Figure 9C(ii)). Similar results were obtained with 5 mM drug-loaded liposomes. The obtained results signify that liposomal Met has superior apoptotic activity via the AMPK/mTOR pathway in breast cancer cells.

### 3.12. In Vitro Tumor Simulation Model

Traditional cell culture studies involve seeding and culturing cancer cells as a monolayer, followed by treatment with a drug or formulation to assess their effectiveness as a treatment. This has served as a reliable tool for evaluating the treatment’s cytotoxic effect on cancerous cells, however, these monolayer studies may not necessarily mimic the tumor conditions [55]. Since tumors exist as a solid mass of cells which proliferate uncontrollably and exponentially, monolayer studies may lack the ability to evaluate the penetration capability of treatment into these solid tumors. Additionally, monolayer treatment may lack the opportunity to investigate the behavioral properties of treatment in a diverse micro-environment as provided by a cancerous mass [37]. Therefore, an in vitro tumor simulation model, also known as a 3D-spheroid cell culture tumor model, has recently been explored due to its ability to mimic in vivo tumor conditions. This approach will help to understand the behavior and effectiveness of treatment in clinically relevant conditions and overcome the limitations presented by monolayer studies. In Vitro studies for Met and Met-encapsulated liposomes were performed via two different sets: i) Single-dose study and ii) multiple-dose study.

#### 3.12.1. Single-Dose Study

**Figure 10:**
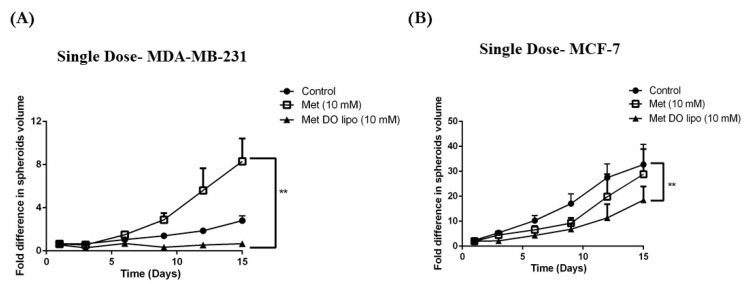
Effect of single-dose treatment on tumorigenic activity of breast cancer cells grown as 3D spheroids simulating conditions of in vivo xenograft tumor growth. (**A**) Quantified analysis of single-dose treatment on MDA-MB-231 spheroids. (**B**) Quantitative representation of anti-tumor activity of treatment expressed as fold difference in spheroid volume versus time. In MDA-MB-231 cells, treatment with Met DO lipo displays significant difference (*p* < 0.01) in spheroid volume growth as compared to Met. Similarly, in MCF-7 cells, significant different (*p* < 0.01 vs. control) can be observed on 15th day after treatment with Met DO lipo, whereas treatment with plain drug (Met) did not show any significant difference. Data represent mean ± SEM (*n* = 6). Scale bar = 100 µm. ** *p* < 0.01.

The single-dose study involves drug treatment only once throughout the 15-day experimental period. This experimental design aids in evaluating the treatment effectiveness by simulating in vitro conditions. Figure 10A demonstrates the fold difference in volume of MDA-MB-231 spheroids (calculated using ImageJ analysis software, Version 1.44). Spheroid volumes at day 1 (prior to treatment) were evaluated and assigned a baseline value of 1 for ease of data presentation. On the 15th day, the MDA-MB-231 control showed a ~2.8 ± 0.5-fold increase in spheroid volume while treatment with Met DO lipo displayed 0.67 ± 0.1-fold difference in spheroid volume from day 1. In comparison, plain Met treatment demonstrated a significant (8.3 ± 2.1-fold; *p* < 0.01) increase in spheroid volume. Therefore, Met DO lipo was shown to effectively display cytotoxic benefits against solid spheroids.

Similarly, in MCF-7 spheroids (Figure 10B), the control group spheroids at the end of 15 days displayed 32.7 ± 8.1-fold difference from day 1. However, treatment with Met DO lipo (10 mM) displayed significant (*p* < 0.01) difference of 18.5 ± 5.4-fold in spheroid volume as compared to control, whereas plain Met treatment (10 mM) did not show any significant difference from the control groups. Similar to MDA-MB-231 triple negative breast cancer (TNBC) cells, Met DO lipo promoted a significant difference in spheroid volumes as compared to Met and the control treated groups. Tumor growth suppression was continually observed until the end of the treatment period (Figure 10A,B).

#### 3.12.2. Multiple-Dose Study

**Figure 11:**
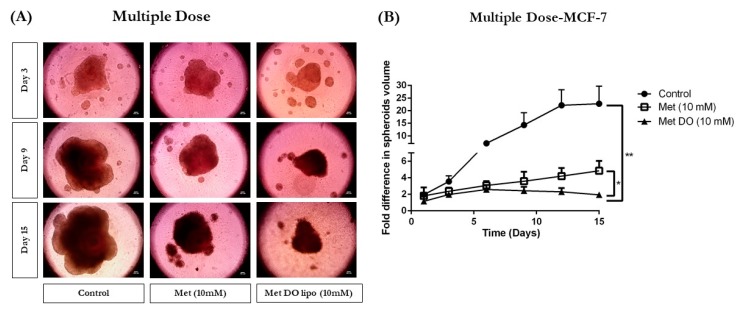
Effect of treatment on tumorigenic activity of MCF-7 cells grown as 3D spheroids simulating conditions of in vivo tumor. (**A**) Images of spheroids demonstrating a difference in size at days 3, 9, and 15 after multiple dose treatment. (**B**) Quantitative representation of anti-tumor activity of treatment expressed as fold difference in spheroid volume versus time for multiple-dose treatment. The multiple-dose treatment with Met DO lipo after 15 days resulted into significant difference (*p* < 0.01) compared to control and *p* < 0.05 versus plain drug (Met) itself. Data represent mean ± SEM (*n* = 6). Scale bar = 100 µm. * *p* < 0.05 and ** *p* < 0.01.

The multiple-dose study was performed to evaluate and mimic the physiological conditions a treatment regimen may experience due to the drug metabolism and clearance from the body, which may require several dosages during the treatment regimen [56]. Briefly, after overnight incubation, the spheroids were treated with control, Met (10 mM), and Met DO lipo (10 mM), followed by removal of 100 µL of media, and replenishment with 100 µL of the respective treatment every three days. This served as a model to provide multiple treatments over a period of 15 days. Figure 11A illustrates representative images (3, 9, and 15 days) of spheroids where it is visibly evident that Met DO lipo resulted in enhanced spheroid growth suppression as compared to plain Met and control throughout the experimental period. Image analyses were performed using the ImageJ software Version 1.44. This analysis revealed significant tumor suppression as shown in Figure 11B.

Met DO lipo demonstrated a 2.1–10.3-fold difference in spheroid volume as compared to plain Met and the control over 15 days. In terms of spheroid volume, Met DO lipo treatment resulted in a 2.1 ± 0.5-fold increase in tumor volume as compared to a 22.8 ± 7.0-fold increase in the control group, as well as a 4.4 ± 1.1-fold increase in plain Met group, thereby demonstrating significantly superior efficacy (*p* < 0.05) (Figure 11B). These results signify the potential of Met DO lipo in providing enhanced protection against tumor progression in breast cancer and provide an excellent insight for expected in vivo behavior in preclinical studies. These results support the hypothesis that liposomal delivery carrier enhance anti-tumor activity of Met which can further be extended in large preclinical studies.

## 4. Discussion

Met, an oral anti-hyperglycemic agent, is the most prescribed drug for type-2 diabetes treatment worldwide [57]. Met has also shown an ability to ameliorate severity of several other diseases including breast cancer, and is said to improve longevity and health-related quality of life [22,58]. Several population studies have demonstrated that diabetic patients receiving Met have a significantly reduced risk of cancer-related mortalities [14,21,59]. Nanomedicine has provided advancements in drug delivery through the fabrication of carriers with desired properties resulting in improved therapeutic efficacy. The success story of Doxil^®^, a liposomal delivery of doxorubicin HCl, has extended the applicability of nanocarriers to a clinical setting [60,61]. In this study, we evaluated Met encapsulation in liposomal vesicles along with cellular uptake, in vitro cellular proliferation, and inhibition of metastatic activity on breast cancer cells. We also studied the effect of a developed formulation in in vitro tumor simulation studies, which mimic the conditions of a tumor microenvironment. Herein, our primary goal for designing Met-loaded liposomes was to reduce the dose of Met required for anti-tumor activity along with providing enhanced therapeutic efficacy at low doses.

The increased anticancer effect shown by liposomal carriers is usually attributed to a nanometer range size of liposomal vesicles (~100 nm), which is vital for increased tumoral accumulation and distribution of liposomal carriers due to an enhanced permeation and retention (EPR) effect [62]. Results obtained from cytotoxicity studies corroborated the fact that Met DO lipo possess the potential to be an effective delivery system for treating breast cancer by showing therapeutic benefits at reduced doses. We have also performed cytotoxicity studies of blank DOTAP liposomes on normal cells such as LLC-PK1 and HEK-293 demonstrating its tolerability and safety. The primary mechanism of any positively charged nanocarriers to augment cytotoxicity of a chemotherapeutic agent is by enhancing the intracellular uptake of the encapsulated drug. While a positive charged component of the nanocarriers may induce oxidation/peroxidation of cell membrane lipoproteins and glycocalyx, the positive charge induced cytotoxicity should be immediate (not molecular pathway/cell cycle dependent), and the presented data precisely reflect the inability of blank (non-drug) DOTAP liposomes in inducing any cytotoxicity in either cancer or normal cells.

Breast cancer is known to be aggressively invasive and metastatic, accounting for ~30% of early-stage cancers to invade locally in adjacent tissues and metastasize to organs such as the liver and lungs [63]. Therefore, in addition to treatment of a localized solid tumor, it is important to focus on controlling the metastatic properties of breast cancer. Therefore, we evaluated the effects of Met DO lipo on breast cancer cell metastasis by performing a cell migration assay. The results demonstrated the effectiveness of the liposomal formulation to inhibit migration by displaying a 2-fold improvement as compared to the drug itself in inhibiting cell migration. We then utilized a clonogenic assay to investigate the effect of Met DO lipo on the stemness of cancer cells, an important phenomenon for metastasis. The assay results demonstrated the efficacy of Met DO lipo in inhibiting cell survival resulting in a profound reduction in the number of colonies as compared to Met and control. This further demonstrated the ability of Met DO lipo to inhibit metastasis of breast cancer cells. Met is reported to induce apoptosis in breast cancer cells by increasing caspase-3 enzymatic activity. We determined caspase-3 enzymatic activity in breast cancer cells after a 6 h treatment, resulting in a significant increase in caspase-3 activity with liposome treatment. This mechanism suggests that the increased cytotoxicity of Met DO lipo may be due to increased apoptosis. Among several underlying mechanisms of apoptotic activity for Met against breast cancer, the most common is the AMPK/m-TOR pathway. Met affects mitochondrial respiration and therefore impacts the ratio of ATP/AMP due to an decrease in ATP levels, ultimately triggering the activation of AMPK [22,32,64]. Upon activation, AMPK downregulates m-TOR, which plays a central role in regulating the energy consuming cellular processes and cellular growth. Enhanced phosphorylation of AMPK and inhibition of p-mTOR then results in the inhibition of cellular proliferation and growth [32,65]. In order to understand the effect of Met DO lipo on these cellular markers of breast cancer cells, western blot analysis was performed. Met DO lipo displayed significantly (*p* < 0.05) superior upregulation of p-AMPK and inhibition of p-mTOR as compared to Met.

The in vitro assays performed may or may not necessarily translate well to in vivo experiments due to the complexity of in vivo tumor models and their uncontrolled cell growth, migration, and invasion causing angiogenesis and varied tumor microenvironment [56,66]. Therefore, we decided to perform in vitro tumor simulation studies wherein solid tumors were grown in specially designed 96-well plates to quantify the effect of Met DO lipo on breast cancer. As discussed earlier, Met DO lipo was found to be effective in suppressing tumor growth significantly as compared to Met and the control in both treatment approaches. Therefore, it can be suggested that Met DO lipo is a promising nanotechnology-based approach, which is proven to be effective in producing desired therapeutic outcomes at significantly reduced doses.

The delivery of Met for breast cancer treatment has been previously reported in only a few studies. Farajzadeh et al. co-encapsulated Met and curcumin in PLGA/PEG nanoparticles demonstrating the growth of breast cancer. While the study was successful in demonstrating the synergistic effect of Met and curcumin, it was reported that Met itself did not show any significant anti-tumor activity against breast cancer [67]. In another study designed to deliver Met alone in PLGA/PEG nanoparticles, while displaying enhanced effect on growth and telomerase reverse transcriptase, the IC_50_ value for both Met and nanoparticles loaded with Met was more than 5 mM. This increased dose requirement for killing 50% cells will directly impact the translational value of formulation to clinical setting.

To our knowledge, this is the first study of encapsulating Met in liposomal delivery carrier for the treatment of breast cancer. Our findings have proven that liposomal Met resulted in a 5-fold reduction in the IC_50_ dose for breast cancer cells as compared to Met resulting in a dose reduction. In addition, supporting evidence from various in vitro studies demonstrate the superior anti-tumor activity of Met loaded liposomes for the treatment of breast cancer.

## 5. Conclusions

The present study acknowledges the idea of repositioning metformin for the treatment of breast cancer and delivering it by liposomal formulations. This idea is supported by extensive in vitro studies, where both Met and drug-loaded liposomes were evaluated for their therapeutic and mechanistic effect in breast cancer cells MDA-MB-231 and MCF-7. Furthermore, liposomes were modified to have a surface positive charge, as they provide improved internalization into the cells as compared to anionic liposomes. The improved internalization in turn provides enhanced therapeutic benefits as more drug is available to exert its chemotherapeutic action. This study lays solid groundwork to build on and pursue the development of Met loaded liposomes in pre-clinical and eventually a clinical setting. Our findings also suggest that encapsulation of Met in cationic lipid carriers addresses and overcomes certain limitations of Met like significant reduction of dose, along with establishing solid chemotherapeutic activity of the drug. In the future, we are planning to perform surface modification of liposomes, which can be exploited for development of targeted liposomes with enhanced specificity and thus better therapeutic efficacy. Furthermore, we will evaluate the efficacy of metformin encapsulated liposomes in A549 xenograft mouse model. To the best of our knowledge, this study is one of a kind and we can say with confidence that liposomal delivery of Met has tremendous potential to evolve as a standalone therapy for breast cancer treatment.

## Figures and Tables

**Table 1:** Physicochemical characterization of developed liposomal formulations. Data represent mean ± SD (*n* = 3).

Formulation	Loading Method	% Drug Entrapment	% Drug Loading	Avg. Particle Size (nm)	Poly-Dispersity Index	Zeta Potential (mV)
Met pass	Passive	5.2 ± 0.2	0.9 ± 0.03	136.3 ± 3.6	0.2 ± 0.1	1.2 ± 0.1
Met pH 3	Active; pH 3.0	24.9 ± 3.9	4.4 ± 0.7	129.6 ± 10.1	0.2 ± 0.1	−2.6 ± 0.8
Met pH 9	Active; pH 9.0	9.9 ± 1.6	1.8 ± 0.3	136.3 ± 4.1	0.2 ± 0.1	0.6 ± 0.3
Met ND lipo	Drug loaded film	58.2 ± 4.5	19.4 ± 1.5	102.3 ± 1.1	0.2 ± 0.0	−1.2 ± 0.9
Met DO lipo	Drug loaded film	65.6 ± 5.2	21.9 ± 1.7	98.2 ± 2.9	0.2 ± 0.0	39.9 ± 1.1

**Table 2:** Release curve fitting details of Met encapsulated liposomal formulations using various models.

Release Model	Met ND lipo		Met DO lipo	
	Equation	*r* ^2^	Equation	*r* ^2^
Zero-order	*Q* = 0.1629 *t* + 0.2689	0.7901	*Q* = 0.1597 *t* + 0.2282	0.8324
First order	ln(1−*Q*) = −0.3142 *t* + 2.0205	0.9989	ln(1−*Q*) = −0.268 *t* + 2.0053	0.9984
Higuchi	*Q* = 0.4673 *t*^1/2^ + 0.0458	0.9546	*Q* = 0.45 *t*^1/2^ + 0.0197	0.9712
Korsmeyer-Peppas	ln*Q* = −1.0357 ln*t* + 1.66	0.9300	ln*Q* = −1.2276 ln*t* + 1.6213	0.9300

*r*^2^, correlation coefficient; *Q*, cumulative drug release and *t*, time.
